# Successful Catheter-Directed Venous Thrombolysis in an Ankylosing Spondylitis Patient with Phlegmasia Cerulea Dolens

**DOI:** 10.5812/iranjradiol.11748

**Published:** 2013-05-20

**Authors:** Hadi Rokni Yazdi, Nematollah Rostami, Homa Hakimian, Mehdi Mohammadifar, Mahsa Ghajarzadeh

**Affiliations:** 1 Department of Radiology, Advanced Diagnostic and Interventional Radiology Research Center (ADIR), Imam Khomeini Hospital, Tehran University of Medical Sciences, Tehran, Iran; 2 Department of Internal Medicine, Modarres Hospital, Shahid Beheshti University of Medical Sciences, Tehran, Iran; 3 Brain and Spinal Injury Research Center (BASIR), Tehran University of Medical Sciences, Tehran, Iran

**Keywords:** Spondylitis, Ankylosing, Thrombosis, Treatment, Venous

## Abstract

Ankylosing spondylitis (AS) is an inflammatory rheumatic disease. Phlegmasia cerulea dolens is a severe form of deep vein thrombosis characterized by swelling, pain, and bluish discoloration. Treatment delay may cause venous gangrene, tissue ischemia, limb loss or death. Here, we present an AS case who presented with phlegmasia cerulea dolens and treated by catheter-directed thrombolysis.

## 1. Introduction

Ankylosing spondylitis (AS) is an inflammatory rheumatic disease in which deep vein thrombosis (DVT) is a rare complication. Phlegmasia cerulea dolens is a severe form of deep vein thrombosis characterized by swelling, pain and bluish discoloration, in which treatment delay would lead to venous gangrene, tissue ischemia, limb loss or death (1, 2). Systemic anticoagulation or thrombolytic therapy, surgical thrombectomy, and catheter-directed thrombolysis are among current therapies in such cases (3).We report a case of AS presented with phlegmasia cerulea dolens, who was successfully treated with catheter-directed thrombolysis and venoplasty.

## 2. Case Presentation

A 41-year-old man, a known case of AS for nine years, was referred to the emergency department with diffuse painful swelling of his left lower limb and scrotum since two weeks prior to admission. He had a history of intermittent mild left lower limb extremity edema in the recent ten months, but no history of a long trip or any recent immobility or hospital admission. Furthermore, no recent trauma history was mentioned and he had no documented history of a previous DVT. Examination of the left lower limb showed diffuse tender swelling in the thigh with a bluish discoloration, while arterial pulses were normal. Factor V leiden, anticardiolipin antibody, serum homocysteine and protein C level were normal in the laboratory evaluation and lupus anticoagulants were negative, but protein C level was prominently reduced. He was under treatment with indomethacin, prednisolone, methotrexate and omeprazole since the beginning of the disease. A Doppler ultrasound demonstrated deep venous thrombosis involving the left common iliac, external iliac and all left lower extremity veins. The diagnosis of phlegmasia cerulea dolens was made based on clinical findings. Systemic anticoagulation therapy was established on the day of admission (500 IU, IV heparin/hour) followed by systemic thrombolysis with 50 mg of rtPA (Actylise, Boehringer, Germany), but no response to treatment was noted. The patient was referred to our department for catheter directed thrombolysis. Before intervention, he filled the informed consent form. Because of kyphosis and joint deformities of prolonged AS, the patient could not tolerate complete prone positioning; therefore, the patient was placed in the semi-prone position on the angiographic table. Subsequently, the left popliteal venous access was obtained under sonographic guide and 6-French arterial sheath (Cordis, USA) was inserted (Figure 1). The venogram showed thrombosis of the popliteal, superficial femoral, common femoral, external and common iliac veins and a long segment stenosis in left external and common iliac veins (Figure 2). Then a 5-French catheter (Multipurpose A1catheter Cordis, USA) over a guide wire (Cordis, USA) was passed through the thrombosis up to the inferior vena cava. Then the catheter was exchanged with a 5-French infusion catheter (Infumed, Angiodynamics, USA) and continuous infusion of rtPA (Actylise, Boehringer, Germany) was performed with dosage of 2 mg/hour through the infusion catheter for 25 hours. Post-thrombolysis venogram revealed partial re-canalization of the veins, but severe stenosis of external and common iliac veins remained (Figure 3).

**Figure:**
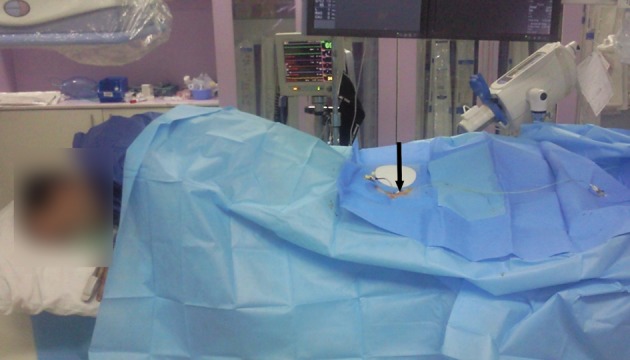
Figure 1. A 41-year-old man, known case of ankylosing spondylitis, semi-prone on the angiographic table. Under sonographic guide a 6 french sheet was inserted in his left thrombosed popliteal vein (arrow)

**Figure:**
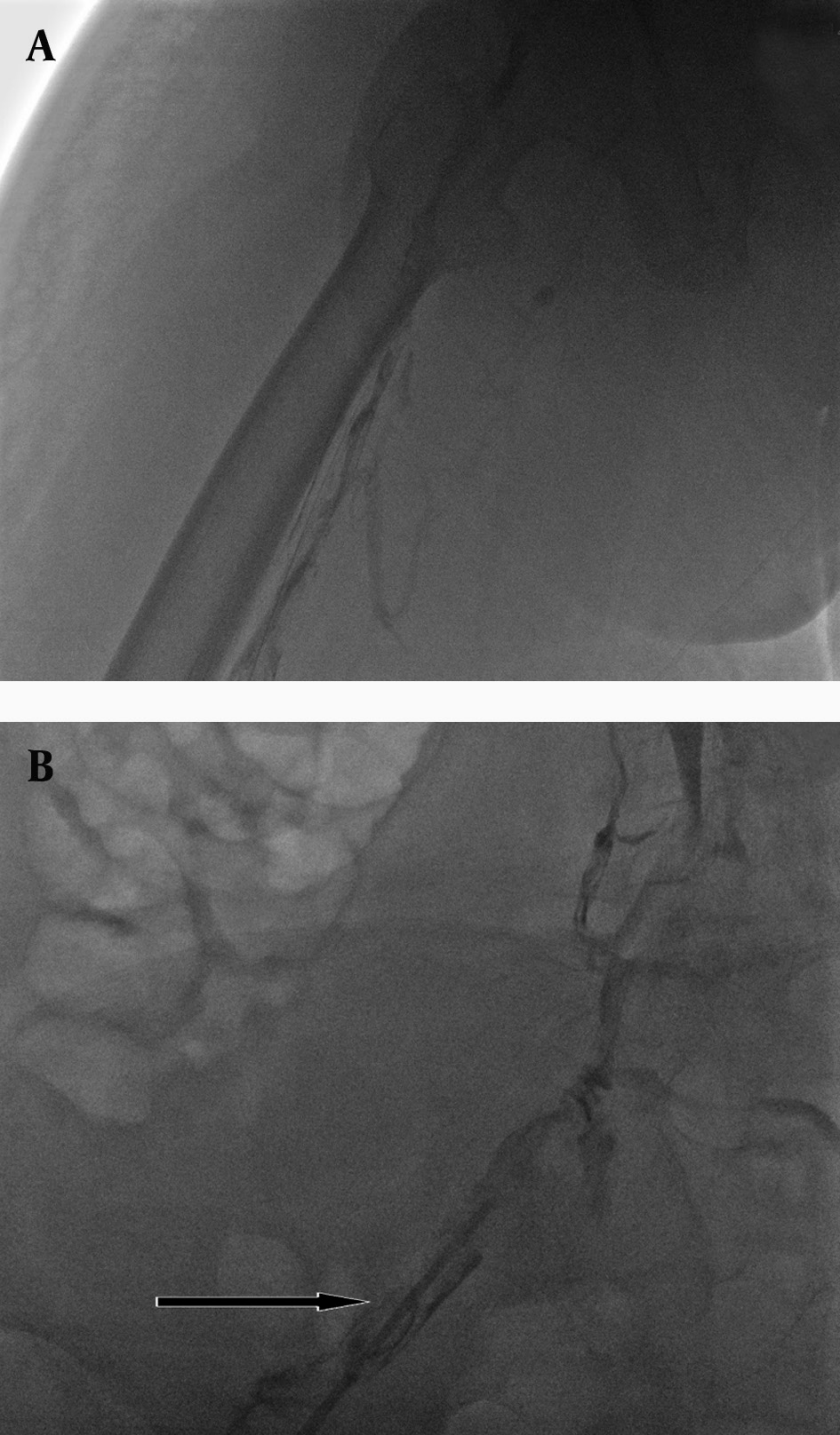
Figure 2. Venography via the popliteal sheet A, Shows diffuse thrombosis of all lower extremity veins as multiple filling defects. B, Catheter injection in the external iliac vein shows long segment stenosis in iliac veins (arrow)

**Figure:**
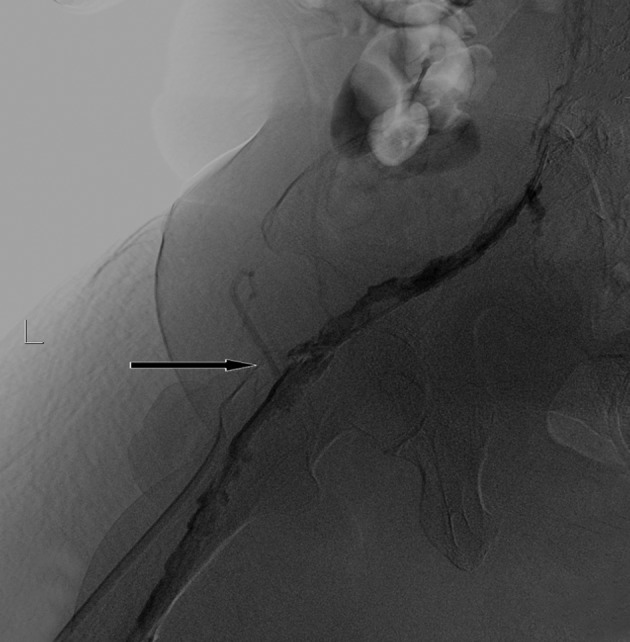
Figure 3. Residual thrombosis (arrow) and severe stenosis of iliac veins after 25 hour catheter directed thrombolysis

A 5-French multipurpose At catheter over a guide wire was then passed through the stenosis of the common iliac vein. First, the tight stenosis was dilated with a 4×20 mm balloon (Cordis, USA) to have enough space to place the larger balloon. Then the stenosis was dilated by a 12×20 mm balloon (Cordis, USA). A 12×90 mm self-expandable stent (Wallstent, Boston Scientific, USA) was deployed in the stenotic segment of the vein. Residual stenosis in the distal part of the stent was dilated with a 12×20 mm balloon (Cordis, USA) (Figure 4). Control venography was normal and patent stent was detected (Figure 5). Significant reduction of swelling was noted one day after the procedure and the patient’s pain and skin discoloration completely disappeared within 48 hours. No complication was noted during and after thrombolysis. So the patient was discharged on daily plavix, aspirin and warfarin. He had no complaint until two months after intervention. After two months, a bilateral non-painful lower extremity edema was noted in both tights. Color Doppler sonography, venography and abdominal CT scans did not reveal DVT or external pressure on the venous system. Total serum protein level was 4 g/L and albumin level was 1.5 g/L due to probable protein loss. Intravenous albumin was administered and all signs disappeared.

**Figure:**
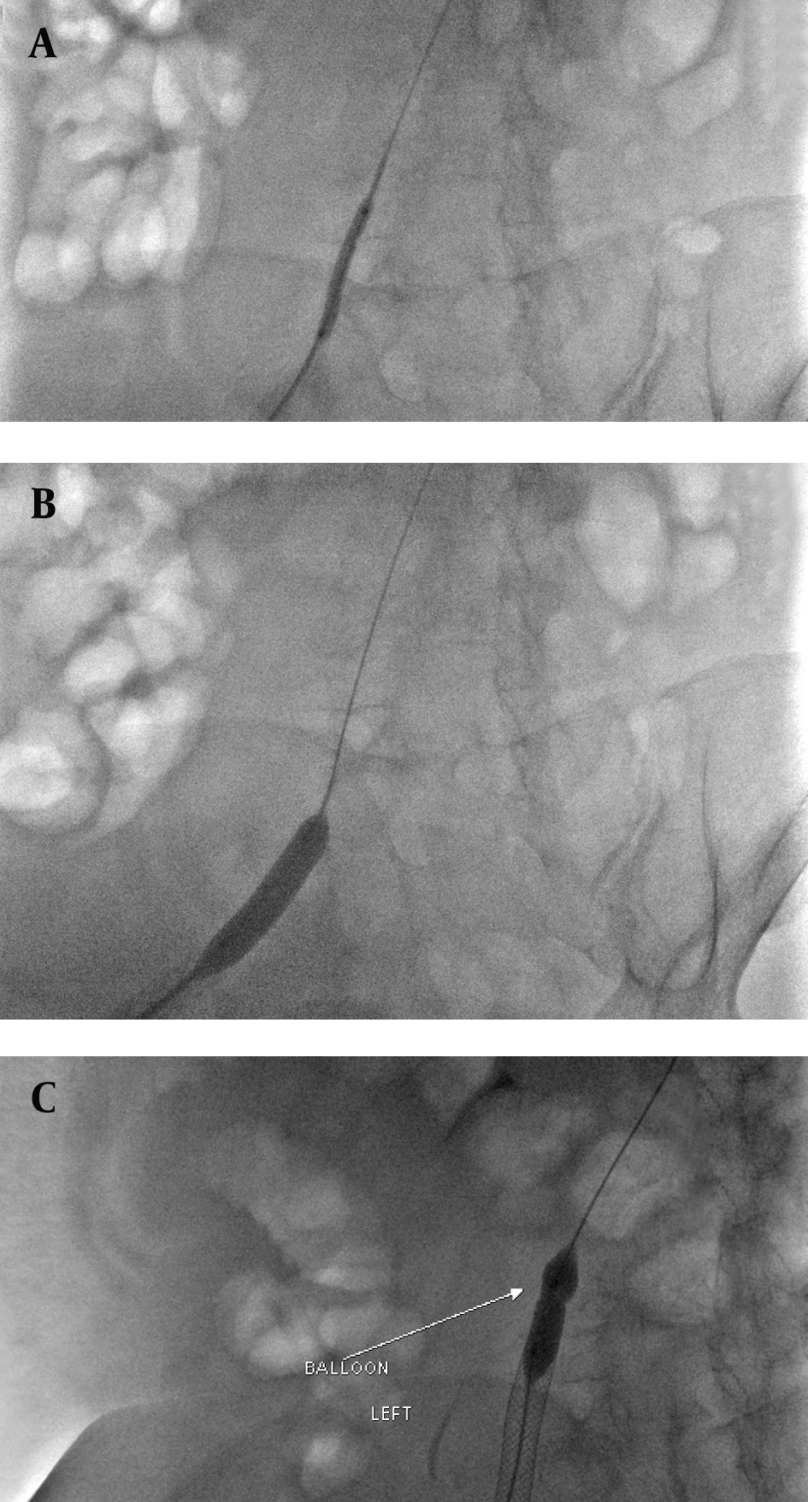
Figure 4. The iliac stenosis A, was first negotiated with a 4×20 mm balloon and then B, dilated with a 12×20 mm balloon consequently C, the 12×90 mm wallstent was deployed and residual stenosis in the distal part of the stent was dilated with the balloon.

**Figure:**
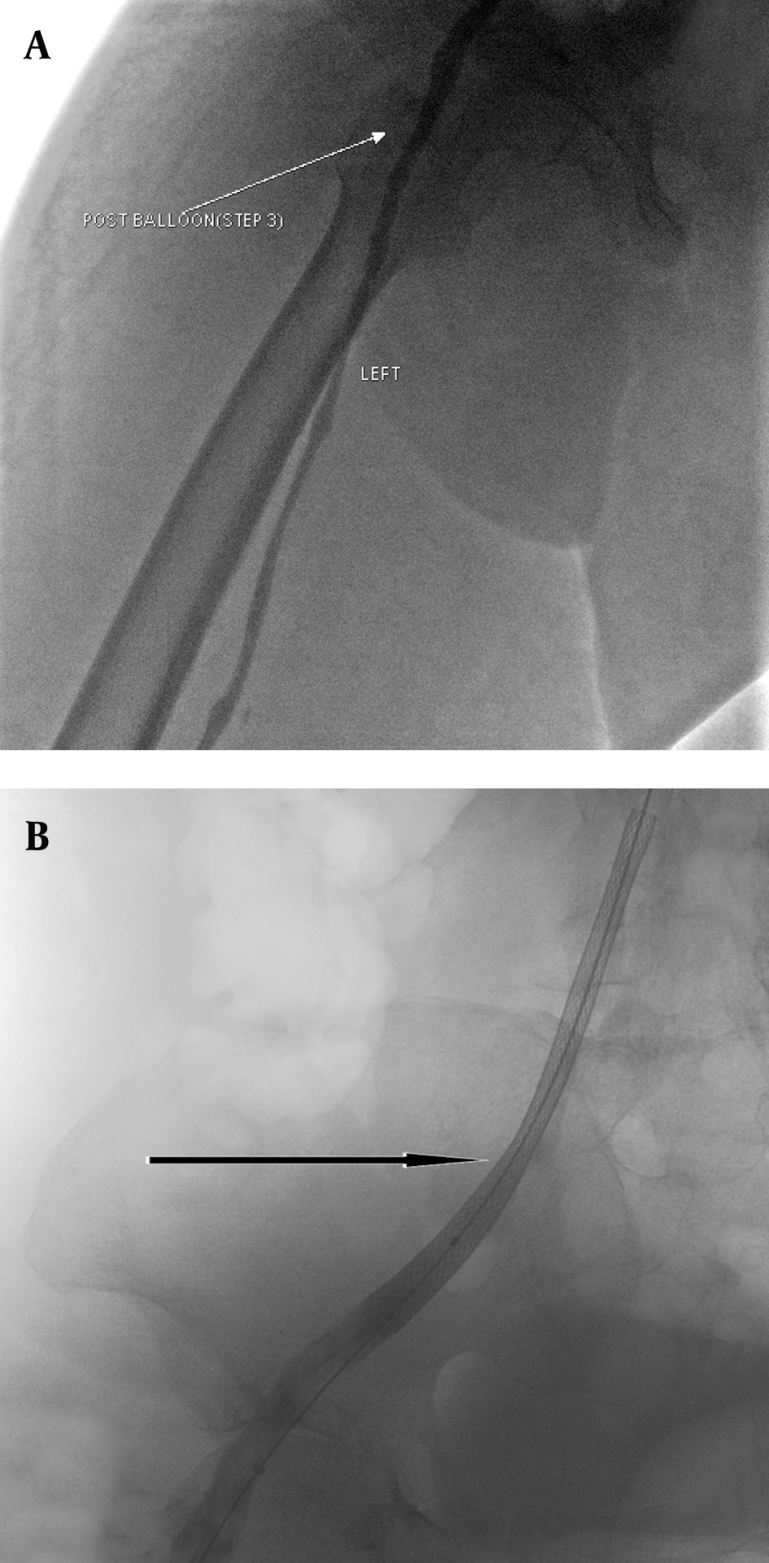
Figure 5. Venogram through the popliteal sheet A, shows complete resolving of the thrombi and the stenosis, B, the stent is in its appropriate position (arrow)

## 3. Discussion

AS is a chronic inflammatory rheumatic disease that involves entheseal sites. Extra-articular manifestations of AS consist of cardiovascular, pulmonary, neurologic and renal involvements. Unlike Behçet’s disease, DVT rarely occurs in AS cases. Phlegmasia cerulea dolens is the result of complete obstruction of the venous drainage that occurs three times more in the left leg compared to the right leg (3). Prevention of thrombosis recurrence, protection of venous valve function, and restoration of blood flow are the main goals of phlegmasia cerulea dolens treatment. Common initial management involves systemic anticoagulation, leg elevation, and compression stockings, but in cases such as our patient, the failure rate is high with this approach (4). So other modalities should be applied. Previous studies showed that even though systemic thrombolytic therapy is more successful than heparin therapy, (as it decreases the propagation of the thrombus and risk of pulmonary embolization), limitation to reach optimal concentration in the thrombus site is its major restriction (5). Patients are at higher risk of post-thrombotic syndrome and venous valvular dysfunction after this therapeutic method (6, 7). Surgical treatment leads to blood flow in a short time, and the risk of recurrent thrombosis and post-thrombotic syndrome disadvantaged its application (8). It is the treatment of choice in cases with acute DVT or cases with complete lumen occlusion. This method is absolutely contra-indicated for patients with progressive malignancy, isolated venous thrombosis of the lower limb, or intolerance to general anesthesia, and is relatively contra-indicated for patients with thrombosis more than 14 days, elderly patients older than 70 years and cases with pulmonary embolism (9). No need for IVC filter placement before thrombectomy is the advantage of this method (9). Catheter-directed thrombolysis was introduced in 1994 by Semba and Dake to treat 21 patients with acute DVT (10). They reported complete lysis in 72%, partial lysis in 20%, and a technical success rate of 85%. They encountered no major complications in their cases. In this method, via the popliteal vein or the tibialis posterior vein, a guide wire was inserted across the thrombus segment that allowed direct delivery of thrombolytic agent into the thrombus. This method, activating plasminogen in the thrombus, would be more successful in thrombus removal than systemic therapy. In patients with extensive stenosis, balloon angioplasty alone or in combination with stent replacement can be performed (3). It is not necessary to place IVC filter in advance (11, 12). Mewissen et al. treated 221 iliofemoral and 79 femoropopliteal DVT cases by means of catheter-directed thrombolysis and urokinase infusions. They demonstrated complete lysis in 31%, 50%-99% lysis in 52%, and less than 50% lysis in 17% of their cases, while at the end, 33% of the patients needed stent replacement. They reported excessive blood loss in 11% and retroperitoneal bleeding in 1% of the patients. Two of six patients who developed pulmonary emboli died (13). Kim et al. used catheter-directed thrombolysis in 178 patients (61 with and 117 without cancer). Major bleeding occurred in three (4.9%) patients. Pulmonary embolization occurred in 1.6% of patients with cancer and 1.7% of patients without cancer (14). Previous case reports showed successful results of catheter-directed thrombolysis in the treatment of phlegmasia cerulea dolens. Two cases with ovarian cancer, who presented with phlegmasia cerulea dolens, were treated successfully with catheter-directed thrombolytic therapy in a study by Tung et al. (3). Lin et al. also found catheter-directed thrombolysis an appropriate method for endoluminal re-canalization in an ovarian cancer patient with phlegmasia cerulea dolens presentation (15). Other studies revealed complete return of arterial pulses and limb rescue after catheter-directed thrombolysis in patients with phlegmasia cerulea dolens (16-18). Our case was successfully treated by this method and showed no complication or adverse effects after intervention, although neurologic events, bleeding, hematoma, fever, nausea, vomiting and death are among procedure-related complications (13). A systematic review conducted by Casey et al. concluded that comparing surgical treatment in cases with acute iliofemoral deep vein thrombosis with systemic thrombolysis, thrombectomy was associated with significant reduction in post-thrombotic syndrome and venous reflux. While comparing systemic thrombolysis with catheter-directed thrombolysis, catheter directed thrombolysis was associated with significant reduction in the risk of post-thrombotic syndrome and venous obstruction (19). We can conclude that catheter-directed thrombolysis is an effective alternative way for clot lysis in AS cases presented with phlegmasia cerulea dolens due to low complications, more effective lysis and better lysis monitoring.
